# Supplementary material for: Deletion of wfs1 Impairs Oligodendrocyte Precursor Cells Dorsal Distribution and Myelination Through the wfs1-hmgcs1 Axis in Zebrafish
Source: Biology (Basel). 2026 Aug 16;15(16):1402. doi: 10.3390/biology15161402 (PMC13510151; doi:10.3390/biology15161402)
Supplement: Supplementary file 1 [file biology-15-01402-s001.zip › File S1-Transcriptome sequencing data and differentially expressed genes (DEGs) in wfs1-- zebrafish at 5 dpf/2.KEGG/wfs1KOvsWT/ALL/src/dre00190.html]

dre00190


- dre:573619

- Up regulated genes

573619(9.34031008711337)

- dre:796997

- Down regulated genes

796997\_1(-1.42761585003721)

- dre:447914

- Up regulated genes

447914\_1(7.364607060603)

- dre:393793

- Down regulated genes

393793\_1(-1.79690843797525)
- dre:436768

- Down regulated genes

436768(-1.23885328223204)

- dre:447942

- Up regulated genes

447942(1.89918802415248)

- Down regulated genes

447942\_1(-8.4689696868631)

- dre:359840

- Up regulated genes

359840(3.81608997342262)

- Down regulated genes

359840\_1(-1.77685900658053)
- dre:767750

- Up regulated genes

767750\_2(7.76955022165362) 767750(6.57768162814625)
- dre:359839

- Up regulated genes

359839(1.49784899389719)

- dre:436768

- Down regulated genes

436768(-1.23885328223204)

- dre:393793

- Down regulated genes

393793\_1(-1.79690843797525)

- dre:447942

- Up regulated genes

447942(1.89918802415248)

- Down regulated genes

447942\_1(-8.4689696868631)

- dre:767750

- Up regulated genes

767750\_2(7.76955022165362) 767750(6.57768162814625)

- dre:359840

- Up regulated genes

359840(3.81608997342262)

- Down regulated genes

359840\_1(-1.77685900658053)
- dre:359839

- Up regulated genes

359839(1.49784899389719)

- dre:573619

- Up regulated genes

573619(9.34031008711337)
- dre:796997

- Down regulated genes

796997\_1(-1.42761585003721)

Close
